# 穿孔素基因缺陷原发性噬血细胞综合征的临床特征：单中心回顾性研究

**DOI:** 10.3760/cma.j.issn.0253-2727.2023.07.009

**Published:** 2023-07

**Authors:** 明珠 喻, 林 吴, 嘉 张, 晶石 王, 旖旎 王, 昭 王

**Affiliations:** 首都医科大学附属北京友谊医院血液科，北京 100050 Department of Hematology, Benjing Friendship Hospital, Capital Medical University, Beijing 100050, China

**Keywords:** 穿孔素, 突变, EB病毒感染, 淋巴组织细胞增多症，噬血细胞性, Perforin, Mutation, Epstein-Barr virus infections, Lymphohistiocytosis, hemophagocytic

## Abstract

**目的:**

探讨穿孔素基因缺陷原发性噬血细胞综合征患者的临床特征。

**方法:**

回顾性分析2014年4月至2021年8月首都医科大学附属北京友谊医院确诊的16例穿孔素基因缺陷原发性噬血细胞综合征患者的临床资料，对患者的突变位点、突变类型、家族史、临床表现及预后等进行分析。

**结果:**

共纳入16例患者，男10例，女6例，中位发病年龄为17.5（4～42）岁。共发现16种突变：11种错义突变，1种无义突变，2种移码突变，2种整码突变。所有患者至少含有1个错义突变且为有害突变，其中c.1349C>T(p.T450M)和c.503G>A(p.S168N)是最常见的突变位点。11例NK细胞活性下降，10例穿孔素蛋白表达下降，8例起病时合并EB病毒感染，2例有家族史，4例累及中枢神经系统。11例患者行异基因造血干细胞移植，8例存活，未移植患者的中位生存时间为8（4～18）个月，移植患者的中位生存时间未达到。

**结论:**

应重视成年人中穿孔素基因缺陷原发性噬血细胞综合征的诊断，EB病毒感染可能是这类疾病的诱因，异基因造血干细胞移植对于患者的预后有较大影响。

## Abstract (en)

**Objective:**

This study aimed to investigate the clinical characteristics of patients diagnosed with primary hemophagocytic lymphohistiocytosis（pHLH）associated with perforin gene deficiency.

**Methods:**

We retrospectively analyzed the clinical data of 16 pHLH patients associated with perforin gene deficiency at Beijing Friendship Hospital, Capital Medical University, from April 2014 to August 2021. The mutation sites, mutation types, family history, clinical characteristics, and prognosis of the patients were assessed.

**Results:**

A total of 16 patients, including ten males and six females, with a median onset age of 17.5 years（range: 4–42 years）, were enrolled in this study. Sixteen different mutations were identified, consisting of 11 missense mutations, one nonsense mutation, two frameshift mutations, and two in-frame mutations. All patients harbored at least one deleterious missense mutation, with the most common mutation sites being c.1349C>T（p.T450M）and c.503G>A（p.S168N）. Decreased natural killer（NK）cell activity was observed in 11 patients, reduced perforin protein expression in ten patients, concurrent Epstein-Barr virus（EBV）infection at onset in eight patients, a family history in two patients, and central nervous system involvement in four patients. Eleven cases underwent allogeneic hematopoietic stem cell transplantation（allo-HSCT）, with eight cases surviving. The median survival time of non-transplanted patients was eight months（range: 4–18 months）, while that of transplanted patients was reported as “not reached”.

**Conclusion:**

Emphasizing the diagnosis of pHLH in adults with perforin gene deficiency. In addition, it should be noted that EBV infection can potentially act as a triggering factor in such disease, and allo-HSCT exerts a substantial effect on the prognosis of patients.

原发性噬血细胞综合征（pHLH）是一类具有遗传背景、由过度活化淋巴细胞和巨噬细胞引起的严重且危及生命的炎症反应综合征。目前有17个基因（PRF1、UNC13D、STX11、STXBP2、RAB27A、LYST、AP3B1、SH2D1A、XIAP、NLRC4、CDC42、MAGT1、ITK、CD27、CD70、CTPS1、RASGRP1）突变以及一个常见染色体异常（9q21.3-22）明确导致pHLH[1]。穿孔素1（PRF1）基因是最早被发现的诊断pHLH的基因[2]，由PRF1突变引起的pHLH称为家族性噬血细胞综合征2型（FHL2）。穿孔素/颗粒酶途径是自然杀伤细胞（NK细胞）和细胞毒性T淋巴细胞（CTL）杀伤靶细胞的最主要途径。该基因突变时，PRF1的表达、活性及稳定性下降，受损的PRF1无法顺利在靶细胞膜上形成管道，导致攻击细胞对靶细胞的杀伤作用受损，大量炎症因子累积失控，进而导致噬血细胞综合征（HLH）。FHL2在pHLH中占13％～58％[3]–[6]，随着测序技术的发展，越来越多的pHLH被发现，目前国内穿孔素缺陷pHLH多为个案报道，本研究搜集首都医科大学附属北京友谊医院16例PRF1相关pHLH，探索其临床特征，进一步提高对本病的认识。

## 病例与方法

1. 病例：回顾性分析首都医科大学附属北京友谊医院2014年4月至2021年8月收治的16例FHL2患者的临床资料。纳入标准：依据HLH-2004诊断标准[7]，符合FHL2诊断。

2. 研究方法：通过高通量测序或一代测序检测基因突变位点。用Sift和polyphen-2预测致病性。从clinvar、Pubmed数据库检索信息确认相关位点文献报道。快速免疫学方法评估NK细胞活性、穿孔素、颗粒酶、CD107a、MUNC13-4、XIAP和SAP蛋白表达。

3. 随访：采用查阅门诊、住院病历及电话随访方式。随访截止时间为2022年2月1日。中位随访时间16.5（4～101）个月。患者总生存期定义为从患者诊断HLH至因任何原因死亡或随访截止的时间间隔。

4. 治疗方案：所有确诊患者治疗后采用HLH-1994方案（依托泊苷+地塞米松）、HLH-2004方案（依托泊苷+地塞米松+环孢素）或DEP±L方案（脂质体阿霉素+依托泊苷+甲泼尼龙±门冬酰胺酶）进行诱导化疗，有合适供者的患者后续行异基因造血干细胞移植（allo-HSCT）。

5. 疗效：参照美国中西部协作组制订的疗效评价标准[8]，主要指标包括血细胞计数、甘油三酯、sCD25、铁蛋白、噬血现象、意识（有中枢神经系统症状的HLH患者）。完全缓解（CR）定义为上述所有指标恢复至正常范围。部分缓解（PR）定义为≥2项临床症状/实验室指标改善25％以上，部分指标需达到以下标准：①sCD25水平下降1/3以上；②铁蛋白和甘油三酯下降25％以上；③不输血情况下，中性粒细胞计数<0.5×10^9^/L者需增加1倍并>0.5×10^9^/L；中性粒细胞计数（0.5～2.0）×10^9^/L者需增加1倍并恢复正常；④ALT>400 U/L者，需下降50％以上。无效（NR）定义为未达到上述标准。

6. 统计学处理：采用描述性统计分析，计数资料用例数表示，计量资料用中位数（范围）表示。

## 结果

1. 一般资料：16例FHL2患者的PRF1突变信息及其临床特征见表1（部分病例在既往研究中报道过[9]）。16例患者中位发病年龄为17.5（4～42）岁，男10例，女6例。患者起病时可有发热（16例）、血小板计数降低（<100×10^9^/L）（12例）、中性粒细胞计数降低（<1.0×10^9^/L）（8例）、贫血（HGB<90 g/L）（5例）、脾大（14例）、甘油三酯升高（>3.0 mmol/L）（2例）、纤维蛋白原降低（<1.5 g/L）（4例）、铁蛋白升高（≥500 µg/L）（11例）、sCD25升高（>6 400 pg/ml）（14例）、骨髓可见噬血现象（12例）、NK细胞活性下降（11例）、中枢神经系统受累（4例）。2例患者有家族史（例4、例5），均为儿童时期发病且死于疾病进展。例4的2个哥哥幼时死于pHLH（具体基因突变未知），1个姐姐具有相同突变位点，诊断为HLH且死亡（图1A）。例5的弟弟具有相同突变位点且发病（图1B）。8例患者起病时合并EB病毒（EBV）感染，3例进行EBV感染亚群检测，均只累及B淋巴细胞。3例同时合并巨细胞病毒（CMV）感染，1例合并人疱疹病毒7型（HHV-7）感染。

**表1:** 16例穿孔素基因突变相关原发性噬血细胞综合征患者的突变位点及临床特征

例号	发病年龄（岁）	性别	突变类型	突变位点	家族史	EBV	CNS	NK细胞活性（%）^e^	穿孔素表达水平（%）^f^		CD107a表达水平（%）^g^		allo-HSCT	结局
									NK细胞	CTL	NK细胞	CTL		
1	6	男	纯合	c.1066C>T(R356W)^a^	无	有	无	0.55	62.00	低于检测下限	18.14	7.20	是	生存
2	18	男	纯合	c.1349C>T(T450M)^a^	无	无	无	11.37	ND	ND	ND	ND	是	生存
3	20	男	纯合	c.1349C>T(T450M)^a^	无	无	无	2.45	73.69	14.53	ND	ND	是	生存
4	4	男	杂合	c.984G>A(W328*)^b^、c.1349C>T(T450M)^a^	有	有	无	12.66	10.65	低于检测下限	10.74	2.20	否	死亡
5	4	女	杂合	c.853_855del(p.285del)^c^、c.1349C>T(T450M)^a^	有	无	有	12.99	33.00	低于检测下限	17.02	2.50	否	死亡
6	9	女	杂合	c.394G>A(p.G132R)^a^、c.218G>A(p.C73Y)^a^	无	有	无	13.06	83.00	26.00	11.72	3.20	是	生存
7	11	男	杂合	c.1083_1094del(p.361_365del)^c^、c.172T>C(p.S58P)^a^	无	无	有	10.74	6.93	6.66	ND	ND	是	生存
8	16	女	杂合	c.65delC(p.P22fs)^d^、c.503G>A (p.S168N)^a^	无	无	无	13.83	35.00	36.00	20.17	3.40	是	死亡
9	16	女	杂合	c.853_855del(p.285del)^c^、c.380A>G(p.N127S)^a^	无	有	无	18.38	78.00	51.00	20.05	2.30	是	生存
10	17	女	杂合	c.46C>T(p.P16S)^a^、c.1066C>T(R356W)^a^	无	有	无	11.29	33.00	1.00	10.42	2.30	是	死亡
11	19	男	杂合	c.1349C>T(T450M)^a^、c.182T>C(p.V61A)^a^	无	无	有	12.73	81.57	27.33	27.15	27.50	是	生存
12	20	女	杂合	c.1349C>T(T450M)^a^、c.503G>A(p.S168N)^a^	无	无	无	16.37	81.00	6.00	16.61	3.70	是	死亡
13	27	男	杂合	c.503G>A(p.S168N)^a^、c.66delG(p.C23AfsX28)^d^	无	有	无	14.16	56.00	低于检测下限	40.14	2.10	否	死亡
14	27	男	杂合	c.65delC(p.P22fs)^d^、c.503G>A (p.S168N)^a^	无	无	有	17.23	ND	ND	ND	ND	否	死亡
15	36	男	杂合	c.G133A(G45R)^a^、c.C1228T(R410W)^a^	无	有	无	32.17	83.52	41.29	30.00	3.10	否	死亡
16	42	男	杂合	c.503G>A(p.S168N)^a^、c.664C>A(p.H222N)^a^	无	有	无	18.81	85.00	1.27	23.00	3.80	是	生存

**注** ^a^错义突变；^b^无义突变；^c^整码突变；^d^移码突变；^e^NK细胞活性正常值≥15.11％；^f^穿孔素表达正常值：NK细胞≥81％，CTL≥2％；^g^CD107a表达正常值：NK细胞>10％，CTL≥2.8％；EBV：EB病毒感染；CNS：累及中枢神经系统；CTL：细胞毒性T淋巴细胞；ND：未检测；allo-HSCT：异基因造血干细胞移植

**图1:**
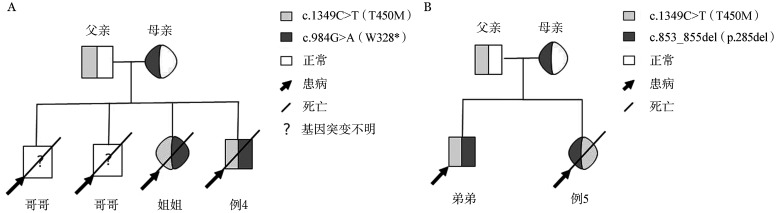
两例穿孔素基因缺陷原发性噬血细胞综合征患者的家系图（A：例4、B：例5）

2. 快速免疫学检测：所有患者均进行NK细胞活性检测（正常值≥15.11％），11例患者低于正常值，其中2例检测值<10％，其余9例检测值>10％。14例进行穿孔素蛋白表达检测（正常值：NK细胞≥81％，CTL≥2％），4例检测正常，10例表达异常，其中4例CTL表达缺失（低于最低检测值）。10例进行CD107a检测（正常值：NK细胞>10％，CTL≥2.8％），6例表达正常，4例CTL表达下降，NK细胞表达均正常。SAP、XIAP、MUNC13-4、颗粒酶表达均正常。

3. 基因突变：16例患者共含有16种突变，11种错义突变，1种无义突变，2种移码突变，2种整码突变。其中3例为纯合错义突变，其余13例均为复杂杂合突变（1例含无义突变和错义突变，3例含移码突变和错义突变，3例含整码突变和错义突变，6例杂合错义突变）。c.1349C>T（p.T450M）是其中最常见的突变（2例纯合，4例复杂杂合），其次是c.503G>A（p.S168N）（5例），其余重复出现的突变包括c.1066C>T（p.R356W）（2例）、c.853_855del（p.285del）（2例）、c.65delC（p.P22fs）（2例）。

4. 治疗及预后：15例患者在初期接受HLH-1994方案、HLH-2004方案或DEP方案治疗，1例未接受治疗。11例达到CR或PR，4例NR。最初缓解的11例患者后期均出现疾病复发，随后接受DEP或L-DEP方案的挽救治疗。共11例患者接受allo-HSCT，基本信息见表2，8例患者存活，3例分别因移植后肺部感染、植入失败、急性移植物抗宿主病死亡。未进行allo-HSCT的5例患者均因疾病进展死亡，未移植患者中位生存时间为8（4～18）个月，移植患者中位生存时间未达到。

**表2:** 11例进行异基因造血干细胞移植的原发性噬血细胞综合征患者基本信息

例号	供者	预处理方案	白细胞植活时间	血小板植活时间	移植后穿孔素表达	结局	生存时间（月）
1	无关供者	Vp 16/Bu/Cy	11 d	13 d	ND	存活	47
2	姐姐	TBI/Cy/Vp 16	10 d	11 d	ND	存活	101
3	母亲	Bu/Cy/Ara-C/Vp 16	22 d	34 d	NK细胞：87.25%，CTL：27.87%	存活	23
6	姐姐	Vp 16/Bu/Cy	13 d	12 d	ND	存活	55
7	姐姐	TBI/Cy/Vp 16	11 d	12 d	NK细胞：28.65%，CTL：0	存活	92
8	父亲	Vp 16/Bu/Cy	13 d	18 d	NK细胞：52.00%，CTL：7.00%	死亡	4
9	弟弟	TBI/Cy/Vp 16	8 d	10 d	ND	存活	70
10	父亲	TBI/Cy	13 d	15 d	ND	死亡	29
11	母亲	Vp 16/Bu/Cy	13 d	21 d	NK细胞：62.97%，CTL：0.97%	存活	15
12	姑姑	Vp 16/Bu/Cy	9 d	20 d	ND	死亡	15
16	妹妹	Bu/Cy/Ara-C/Vp 16	12 d	12 d	ND	存活	7

**注** Vp 16：依托泊苷；Bu：白消安；Cy：环磷酰胺；TBI：全身放射治疗；Ara-C：阿糖胞苷；CTL：细胞毒性T淋巴细胞；ND：未检测

## 讨论

pHLH通常被认为是一种婴幼儿疾病，约90％在2岁以前发病，1952年FARQUHAR等[10]提出，pHLH的发病年龄为9周。近年来，关于pHLH发病年龄的文章有多篇报道[3]–[5],[11]，发病最迟的患者70岁才出现HLH相关症状。FHL2发病年龄推迟考虑存在残余的穿孔素活性及细胞毒性功能，基因突变位点类型与发病年龄相关，亚效突变产生的穿孔素蛋白减少，在迟发型FHL2中多见，拥有两个破坏性突变（插入、缺失、无义突变、框架移码）的患者发病年龄小于只有错义突变的患者。Mhatre等[5]的研究显示，无义突变与NK细胞活性缺失有显著的相关性，且纯合突变的发病年龄比单杂合和复杂杂合突变更早。在本研究中，患者的中位发病年龄17.5（4～42）岁，所有患者均至少含有1个错义突变位点，考虑是延迟发病的主要原因。

病毒感染是HLH常见的诱发因素之一，Cetica等[6]认为，HLH是外源性触发因素和遗传易感性综合作用的结果，当受到病毒感染及其他环境因素影响时，基因中的“温和”突变发挥重要作用。在本研究中，50％患者起病时合并EBV感染，CMV（1例）、HHV-7（3例）为其他合并的病毒感染。Gao等[12]报道了10例EBV诱发的迟发型pHLH，杂合改变和环境因素如EBV感染考虑与迟发型pHLH相关，EBV感染患者多为高病毒载量，EBV的靶细胞多为NK细胞，有时伴有T细胞。在本研究中，3例进行EBV感染亚群检测的患者主要累及B细胞，且起病时EBV载量偏低，在初次治疗后转阴。

pHLH中的基因突变有遗传异质性及种族特异性。PRF1（OMIM，603553）定位于染色体10q21-q22，共包含3个外显子，主要在2、3号外显子编码，拥有MCAPF、EGF样、C2三种主要结构域。至今已报道100多个有害或可能有害位点[2]–[6],[9],[11]–[13]，其中常见位点主要包括c.10C>T（p.R4C）、c.50delT（Leu17fsX34）、c.65delC（p.P22fs）、c.272C>T（p.A91V）、c.503G>A（p.S168N）、c.1090-1091delC（L364fs）、c.1122>4A（Trp374X）、c.1349C>T（p.T450M）等，中国pHLH人群中的高频突变位点有c.1349C>T（p.T450M）、c.1066C>T（R356W）、c.853_855del（p.285del）、c.503G>A（p.S168N）、c.65delC（p.P22fs）。本研究中，c.1349C>T（p.T450M）为最常见的突变位点（6例），北京儿童医院的一项研究（6/10）[3]和国内一项多中心回顾性研究（8/22）[14]也有类似结论。该突变位点位于C2结构域，Feldmann等[15]认为其不影响穿孔蛋白成熟及在CTL细胞中的表达，但与钙的结合能力丧失，使体外CTL活性受损。c.503G>A（p.S168N）的发生率仅次于c.1349C>T（p.T450M）。在既往报道中，c.50delT（Leu17fsX34）在非裔美洲人中常见，c.1122>4A（Trp374X）被认为是土耳其来源，c.1090-1091delC（L364fs）在日本人中是主要的突变类型。经过进一步检索，c.148G>A（p.V50M）、c.445G>A（G149S）、c.673C>T（R225W）在各人群中均出现，c.666C>A（H222Q）、c.658G>A（G220S）主要在高加索人群中报道，c.386G>C（W129S）主要在印度人群中报道，c.207del（p.D70fs）、949G>A（p.G317R）是日本人群常见的位点[3],[5],[9],[11],[13]–[14],[16]–[22]。

目前，allo-HSCT被认为是唯一有望治愈pHLH的手段。本报道显示移植存活率72.7％，未移植患者均死亡。DEP或L-DEP方案在一线治疗方案进展时仍具有较高缓解率，为后期移植提供了机会。依帕伐单抗是一种靶向IFN-γ的单克隆抗体，2018年11月被美国食品药品监督管理局（FDA）批准用于治疗难治、复发、进展性或对常规HLH疗法不耐受的成人和儿童pHLH患者，2022年3月该药在中国获批。Locatelli等[23]报道，70％接受依帕伐单抗治疗的患者可以进行allo-HSCT。阿仑单抗（Alemtuzumab）一线治疗pHLH的前瞻性试验目前正在进行中（NCT02472054）。基因修饰的造血干细胞或外周T细胞可以恢复效应细胞的细胞毒性，也可以保护穿孔素缺乏的小鼠免受淋巴细胞性脉络丛脑膜炎病毒（LCMV）感染[24]–[25]，是一种潜在的治疗穿孔素缺陷所致pHLH的方法。

随着对pHLH认识的加深以及基因测序的进一步开展，越来越多的致病位点被发现，本研究涉及的位点多为错义突变位点，提供了中国FHL2的流行突变位点数据，今后还需进行更大样本量的研究来验证，对于难治复发的成人患者，需警惕穿孔素基因缺陷pHLH可能，病毒感染尤其是EBV感染是其常见诱因。免疫学检测尤其是穿孔素联合CD107a对于FHL2的快速诊断有提示作用，对于已经确诊的FHL2患者，建议尽快行allo-HSCT。未来需要进一步探索致病位点、发病机制及相关危险分层以提高诊断和治疗能力，改善患者的预后。
